# Dyslipidemia profiles and their sex-dimorphic impact on cardiometabolic risk in Arab adolescents

**DOI:** 10.3389/fped.2025.1685636

**Published:** 2025-11-25

**Authors:** Nasser M. Al-Daghri, Hanan A. Alfawaz, Malak N. K. Khattak, Nasiruddin Khan

**Affiliations:** 1 Biochemistry Department, College of Science, King Saud University, Riyadh, Saudi Arabia; 2 Department of Food Science & Nutrition, College of Food & Agriculture Sciences, King Saud University, Riyadh, Saudi Arabia; 3 Department of Health Sciences, College of Applied and Health Sciences, A’Sharqiyah University, Ibra, Oman

**Keywords:** dyslipidemia, CVD, obesity, adolescents, Saudi Arabia

## Abstract

**Purpose:**

Recently, the prevalence of dyslipidemia has been on the rise among Saudi children and adolescents. Although dyslipidemia is a well-established cardiovascular disease (CVD) risk factor, the strength of its associations relative to other cardiometabolic risk factors, particularly in the pediatric population, remains unclear. This study aims to identify the associations of both single and combined lipid abnormalities, obesity status and CVD risk factors among Saudi adolescents.

**Methods:**

This cross-sectional study included 4,930 adolescents [1,773 boys [mean age 14.6 ± 1.6] and 3,157 girls [mean age 14.4 ± 1.6]]. Dyslipidemia was defined based on the National Heart Lung and Blood Institute and National Cholesterol Education Program guidelines for adolescents.

**Results:**

Overall, 46.5% had at least one abnormal lipid profile level, while 18.6% and 2.6% exhibited changes in two or all lipid profile variables, respectively. The most common lipid abnormalities were borderline to high triglycerides and low HDL-C levels. Regardless of gender, higher BMI was associated with more significant changes in lipid profile parameters. The boys with hyperglycemia was found to be significantly associated with more altered combined dyslipidemia than girls.

**Conclusions:**

Dyslipidemia patterns related to obesity are commonly observed in Arab adolescents. Therefore, it is imperative to implement public health interventions that prioritize school-based physical education initiatives and lipid management strategies for this population.

## 1 Introduction

Globally, cardiovascular disease (CVD) stands as a major cause of mortality leading to an estimated 18.6 million of deaths in 2019 as compared to 12.1 million in 1990 (1). Reports from the US suggest CVD as leading cause of death and indicates a continuous increase in its risk factors in the near future (2). The key reason for this unexpected rise in CVD is coupled with increased obesity and cardiovascular risk factors (3). Dyslipidemia is defined as an elevation in total cholesterol (TC), low-density lipoprotein (LDL-C), triglycerides (TG), non-high-density lipoprotein (Non-HDL-C) or decreased high-density lipoprotein cholesterol (HDL-C) (4). Combined dyslipidemia, is a predominant abnormal lipid pattern prevalent in 30%–50% of obese adolescents and specifically characterized by moderate to severe elevation in TG and non-HDL-C with reduced HDL-C (5). Dyslipidemia has been shown to be associated with cardiovascular risk factors [obesity, diabetes mellitus (DM), hypertension (HTN) and smoking] (6). Early atherosclerosis can be detected in childhood and adolescence and its advancement depends on exposure of several risk factors (7). Moreover, atherosclerosis in childhood and adolescence is associated with dyslipidemia, and overweight or obesity (8). Early detection and control of CVD risk factors in childhood and adolescence may prevent and delay the progression of the disease (9).

A recent report demonstrated CVD as the leading cause of death in the Middle East and North African region including Saudi Arabia (10). Moreover, the prevalence of CVD is alarmingly high in Saudi Arabia and is associated with a high rise in CVD risk factors especially obesity, DM, dyslipidemia, hypertension and others (11, 12). Increased sedentary behavior, physical inactivity and unhealthy industrialized diet are becoming a norm in Saudi children and adolescents and are negatively impacting their overall health status (13). In the Gulf Cooperation Council (GCC) countries including Saudi Arabia (SA), Bahrain, Kuwait, Oman, Qatar, and United Arab Emirates, the increased prevalence of obesity is worrisome among adolescents with a prevalence of 20% among male and female ranging from 10 to 19 years old (14).

The global burden of dyslipidemia is on rise and its early detection and prevention might prevent its progression to later stages of life (15). In a recent study, a gender-based prevalence of dyslipidemia and its correlates such as BMI-for- age, serum ferritin and calcium and dietary behavior was demonstrated among adolescents (age 10–19 years) across all the 13 regions of Saudi Arabia. The study also reported sex specific mean lipid levels. However, the combination of lipid profile panel was limited (three profile) (16). Previous scientific evidence about lipid profile parameters conducted within the Saudi population has shown limitations, such as sample size and the inclusion of both adults and children as the target population (17–19). Therefore, to the best of our knowledge, we found no published study that assessed the prevalence, coexistence, and association of eight different lipid profile stratification with BMI status among Saudi adolescents. Therefore, in this cross-sectional study, we aimed to determine the prevalence of individual and combined lipid abnormalities and its association with BMI, and cardiovascular disease risk factors in Saudi adolescents. In addition, the study demonstrates gender based associated risk factors for different altered lipid profiles.

## 2 Materials and methods

### 2.1 Participants and data sources

In this cross-sectional study, 4,930 apparently healthy Saudi adolescents [1,773 boys [mean age 14.6 ± 1.6] and 3,157 girls [mean age 14.4 ± 1.6]] years participated from three different cohorts. The participants were recruited randomly from different households and governmental schools across the city of Riyadh, Saudi Arabia. Prior to the study, each participant submitted an assent form. In addition, parents signed a written consent, as well as answered a general questionnaire containing past and present demographic and medical history. Participants’ data were obtained from three projects as follows: Data from 2010 were collected from the Riyadh Cohort database, where participants were invited door-to door from different households, and assessments were performed at the nearest primary care center (20). Data from 2015 were collected from the Vitamin D Schools Project database, a collaborative study between the Prince Mutaib Chair for Biomarkers of Osteoporosis (PMCO) in King Saud University (KSU) and the Ministry of Education in Saudi Arabia involving 34 schools. The project was registered in the Saudi Food and Drug Administration (SFDA) clinical trial registry (SCTR no. 16012402). The detailed structure of the 2015 study participants was described elsewhere (21). The 2019 participant's data were collected from the diabetes schools project, a collaborative study between the Chair for Biomarkers of Chronic Diseases (CBCD) at KSU and the Saudi Diabetes Charity Association (SDCA), Riyadh, Saudi Arabia, also involving the same schools. Ethical approval was obtained from King Saud University Medical City (KSUMC) (no. E19-4239, Oct 29, 2019).

### 2.2 Anthropometric measurements

The participants were instructed to come to their respective schools in a 10-h overnight fasting state. The visit included an anthropometric assessment of weight (kg), height (cm), waist and hip circumferences (cm) using standard methodology. Weight and height were recorded to the nearest 0.2 kg and 0.5 cm, respectively, using an appropriate international standard scale (Digital Pearson Scale, ADAM Equipment Inc., USA). Waist and hip measurements were done in centimeters using a nonstretchable tape. Blood pressure was measured twice within a 15 min interval using a standardized mercury sphygmomanometer on the right upper arm. The mean systolic and diastolic blood pressure of the 2 measurements taken 15 min apart was noted.

### 2.3 Biochemical analyses

Fasting blood samples were collected by trained nurses. Biochemical analyses, including fasting blood glucose and lipid profile levels were obtained routinely (Konelab, Vantaa, Finland). Reagents for the analysis of lipids were obtained from ThermoFisher Scientific, Waltham, MA, USA. For HDL-C, the precipitating reagent used was phosphotungstate. The LDL-C was calculated using the Friedwald equation [total cholesterol—HDL-C—(triglycerides/2.2)].

### 2.4 Assessment of lipid profile modifications and operational definitions

The evaluation for desirable, borderline/high, and low lipid profile panel parameters were based on the National Heart Lung and Blood Institute and National Cholesterol Education Program guidelines for adolescents: LDL-C ≥ 130 mg/dL, HDL-C < 40 mg/dL, and TG ≥ 130 mg/dL (22). Blood pressure was considered elevated if: ≥90th percentile to <95th percentile, while elevated glucose was defined as fasting blood glucose >100 mg/dL (>5.6 mmol/L) (23). The participants were categorized into eight different lipid profiles: profile 1 (normal lipid profile variables); profiles 2–4 (those with only one abnormal lipid level); profile 5–7 (those with two abnormal lipid variables); and profile 8, (participants with all abnormal variables of the lipid profile).

The BMI z-scores were calculated using the reference values established by the World Health Organization (WHO) (24). Participants in the study were categorized into specific BMI groups based on their respective BMI z-scores. Adolescents whose BMI z-score for their respective sex and age group was less than or equal to ≤–2 were classified as having very low or low weight. Those with z-scores >–2 and <+1 were deemed to have an appropriate weight. A z-score ranging from ≥+1 and <+2, indicated that the individual was overweight, while a z-score of ≥+2 or higher classified them as obese.

### 2.5 Statistical analysis

Data were analyzed using SPSS (version 22 Chicago, IL, USA). Continuous data were presented as mean ± standard deviation (SD) and median (1st and 3rd) Percentile for variables following Gaussian and non-Gaussian variables. Categorical data were presented as frequencies and percentages (%) and associations between checked Chi-square and Fisher Exact test. All continuous variables were checked for normality using Kolmogorov–Smirnov test if not normal transform to log transforms. Differences in groups were analyzed using an independent student test and one-way analysis of variance test for normal variables. Further multinomial logistic regression was used to identify the association of risk factors for lipid abnormalities with HTN, WC and glucose. *P* value <0.05 was considered statistically significant.

## 3 Results

Table 1 represents the general characteristics of the participants including a total of 4,930 adolescents with the mean age of 14.5 ± 1.6 years, with 64% of girls and 36% of boys. A significantly high BMI and WHR were observed in boys (*p* = 0.02, < 0.001, respectively) than girls. Significantly higher proportion of girls were overweight than boys (22.2% vs. 18.8%, *p* < 0.001, respectively), while more boys were obese than girls (20.1% vs. 12.3%, respectively). While comparing the lipid parameters, TC, HDL-C, and LDL-C were significantly higher in girls (*p* < 0.001 for all three variables), while boys showed significantly higher TG level (*p* < 0.001). Systolic BP was significantly high in boys (*p* = 0.002), while diastolic BP was high among girls (*p* < 0.001). Regarding the dyslipidemia status, the prevalence was significantly high among boys demonstrating high triglyceride, low HDL-C (*p* < 0.001 for both), and high glucose (*p* < 0.001), while girls showed higher prevalence with increased WC (*p* < 0.001).

**Table 1:** Clinical characteristics of participants.

Parameters	All	Boys	Girls	*P*-value
*N* (M/F)	4,930	1,773	3,157	
Age (years)	14.5 ± 1.6	14.6 ± 1.6	14.4 ± 1.6	<0.001
Height (cm)	155.6 ± 10.0	158.5 ± 11.6	154.1 ± 8.6	<0.001
Weight (kg)	55.1 ± 16.5	58.1 ± 19.5	53.4 ± 14.4	<0.001
BMI (kg/m^2^)	22.6 ± 5.8	22.8 ± 6.3	22.4 ± 5.5	0.02
BMI Z-score	0.00 ± 1.0	0.043 ± 1.09	−0.024 ± 0.95	0.01
Waist (cm)	71.4 ± 15.1	70.4 ± 20.1	71.9 ± 11.3	<0.001
Hips (cm)	87.6 ± 17.5	80.6 ± 22.5	91.6 ± 12.3	<0.001
WHR	0.82 ± 0.11	0.88 ± 0.10	0.79 ± 0.10	<0.001
Systolic BP (mmHg)	115.8 ± 15.5	116.7 ± 14.4	115.3 ± 15.9	0.002
Diastolic BP (mmHg)	70.9 ± 11.7	68.0 ± 10.3	72.6 ± 12.2	<0.001
Glucose (mg/dL)	92.7 ± 17.3	94.3 ± 17.6	91.9 ± 17.1	<0.001
Total Cholesterol (mg/dL)	162.8 ± 32.9	159.3 ± 30.9	164.8 ± 33.7	<0.001
HDL-Cholesterol (mg/dL)	39.41 ± 10.3	37.7 ± 9.2	40.3 ± 10.7	<0.001
LDL-Cholesterol (mg/dL)	104.1 ± 30.9	101.5 ± 27.6	105.6 ± 32.5	<0.001
Triglycerides (mg/dL)	97.03 ± 44.4	101.3 ± 48.9	94.6 ± 41.4	<0.001
Obesity status				<0.001
Normal	3,151 (63.9)	1,083 (61.1)	2,068 (65.5)	
Overweight	1,036 (21.0)	334 (18.8)	702 (22.2)	
Obese	743 (15.1)	356 (20.1)	387 (12.3)	
Cardiometabolic status				
Elevated BP	1,074 (21.8)	396 (22.3)	678 (21.5)	0.25
Elevated glucose	1,032 (20.9)	444 (25.0)	588 (18.6)	0.01
Abdominal obesity	466 (9.5)	115 (6.5)	351 (11.1)	<0.001
High Triglycerides	873 (17.7)	370 (20.9)	503 (15.9)	<0.001
Low HDL-Cholesterol	2,740 (55.6)	1,101 (62.1)	1,639 (51.9)	<0.001

Data presented *N* (%) and mean ± SD; significant at *p* < 0.05.

### 3.1 Prevalence of dyslipidemia

Approximately 32.3% (*N* = 1,593) of the total adolescents demonstrated normal results across all lipid parameters, in contrast to those who exhibited at least one abnormal lipid profile level (*n* = 2,293, 46.5%). Furthermore, around 18.6% (*n* = 918) and 2.6% (*n* = 126) displayed alterations in two and all lipid profile variables, respectively.

### 3.2 Sex based co-existence of various lipid profile

In the case of boys, 29.5% of adolescents displayed a normal lipid profile, 46.6% had one altered profile, and 21.3% presented at least two altered profiles, whereas 2.7% exhibited alterations across all lipid profiles. Conversely, among girls, 33.9% showed no alterations in their lipid profiles, 46.5% had one altered profile, 17.1% had two altered profiles, and 2.5% demonstrated alterations in all lipid profiles. The most commonly found altered pair of lipid combinations were borderline/high TG and low HDL-C level among adolescents with higher means of BMI and BMI z-score (Table 2).

**Table 2:** Combined changes in 8 different profiles in increasing order of BMI and BMI z-score.

Profile	Triglycerides (mg/dL)	LDL-C (mg/dL)	HDL-C (mg/dL)	*N* (%)	BMI z-Score (mean 95% CI)	BMI mean ± SD
	Desirable <90 Borderline/high ≥130		Desirable <110 Borderline/high ≥130		Desirable ≥45 Low <40	
	All participants					
1	Desirable	Desirable	Desirable	1,593 (32.3)	−0.272 (−0.31 to −0.23)	20.99 ± 4.9
2	Desirable	Borderline/high	Desirable	370 (7.5)	−0.052 (−0.15 to 0.042)	22.26 ± 5.4
3	Borderline/high	Desirable	Desirable	172 (3.5)	0.013 (−0.14 to 0.17)	22.64 ± 5.9
4	Desirable	Desirable	Low	1,751 (35.5)	0.032 (−0.014 to 0.08)	22.75 ± 5.7
5	Desirable	Borderline/high	Low	344 (7.0)	0.221 (0.098 to 0.34)	23.85 ± 6.7
6	Borderline/high	Borderline/high	Desirable	54 (1.1)	0.377 (0.08 to 0.67)	24.75 ± 6.4
7	Borderline/high	Desirable	Low	520 (10.5)	0.413 (0.32 to 0.51)	24.97 ± 6.4
8	Borderline/high	Borderline/high	Low	126 (2.6)	0.653 (0.43 to 0.87)	26.36 ± 7.2
Boys						
1	Desirable	Desirable	Desirable	523 (29.5)	−0.359 (−0.44 to −0.28)	20.49 ± 5.3
2	Desirable	Borderline/high	Desirable	88 (5.0)	0.068 (−0.16 to 0.30)	22.96 ± 6.2
3	Borderline/high	Desirable	Desirable	40 (2.3)	−0.071 (−0.39 to 0.26)	22.15 ± 5.9
4	Desirable	Desirable	Low	697 (39.3)	0.049 (−0.027 to 0.13)	22.85 ± 5.9
5	Desirable	Borderline/high	Low	95 (5.4)	0.431 (0.16 to 0.70)	25.10 ± 7.8
6	Borderline/high	Borderline/high	Desirable	21 (1.2)	0.617 (0.15 to 1.09)	26.14 ± 5.9
7	Borderline/high	Desirable	Low	261 (14.7)	0.504 (0.36 to 0.64)	25.50 ± 6.6
8	Borderline/high	Borderline/high	Low	48 (2.7)	0.872 (0.56 to 1.18)	27.63 ± 6.2
Girls						
1	Desirable	Desirable	Desirable	1,070 (33.9)	−0.229 (−0.28 to −0.18)	21.24 ± 4.6
2	Desirable	Borderline/high	Desirable	282 (8.9)	−0.089 (−0.19 to 0.01)	22.05 ± 5.1
3	Borderline/high	Desirable	Desirable	132 (4.2)	0.039 (−0.14 to 0.22)	22.79 ± 6.0
4	Desirable	Desirable	Low	1,054 (33.4)	0.021 (−0.036 to 0.08)	22.68 ± 5.4
5	Desirable	Borderline/high	Low	249 (7.9)	0.141 (0.008 to 0.27)	23.38 ± 6.2
6	Borderline/high	Borderline/high	Desirable	33 (1.0)	0.224 (−0.175 to 0.62)	23.87 ± 6.5
7	Borderline/high	Desirable	Low	259 (8.2)	0.322 (0.19 to 0.45)	24.43 ± 6.1
8	Borderline/high	Borderline/high	Low	78 (2.5)	0.518 (0.22 to 0.82)	25.58 ± 7.6

Data presented *N* (%), mean ± SD and Mean BMI z score with 95% CI.

**Figure 1:**
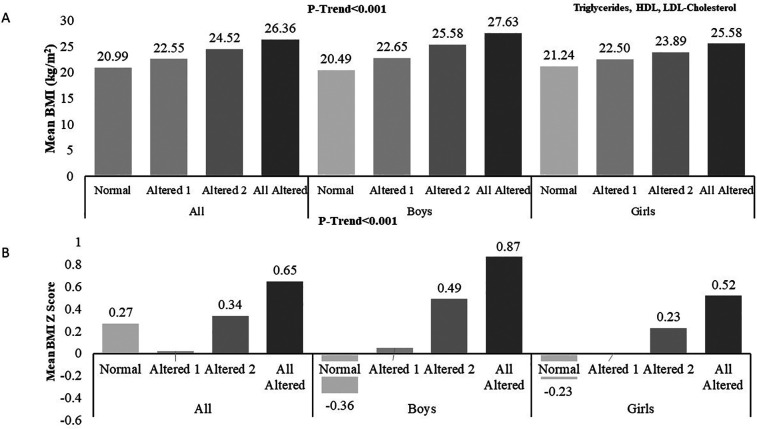
Association of BMI **(A)** and BMI z-score **(B)** with altered lipid variables (triglycerides, HDL-cholesterol and LDL-cholesterol).

Irrespective of sex, an increase in BMI or BMI z -score corresponds to greater changes in lipid profile parameters (Figure 1).

### 3.3 Association of risk factors with lipid profile

Table 3 shows the odds of predicting association of risk factors for combined changes in different lipid profiles using normal lipid level variables as reference among adolescents. Overall, the odds of having greater altered lipid profile parameters (profile 4–8) were more likely to increase several folds with an increase in WC [OR: 5.32, (95% CI:3.42–8.27), *P* < 0.001] and elevated BP [OR: 2.45, (95% CI:1.80–3.32), *P* < 0.001]. However, higher glucose level was found to be significantly associated with one and two altered lipid profiles (profile 2 and 7) parameters [OR: 1.51, (95% CI:1.16–1.96), *P* = 0.002], [OR: 1.53, (95% CI:1.21–1.92), *P* < 0.001], respectively, and no significant statistical difference was observed for profile 8 with all altered lipid parameters. In boys and girls, the increase in WC was associated with increased risk of having borderline-high TG and LDL-C level and low HDL-C level, and emerged as the strongest independent risk factor. The odds of having increased altered lipid profiles parameters (profile 4–8) was higher among boys than girls [OR: 9.95, (95% CI:3.75–26.38), *P* < 0.001] and OR: 3.85, (95% CI:2.31–6.41), *P* < 0.001), respectively. Similarly, irrespective of sex, the risk of having elevated BP was significantly associated with increased co-existence of borderline-high TG and LDL-C level and low HDL-C level. In addition, the likelihood of having hyperglycemia was significantly high among boys associated with borderline-high TG and low HDL-C level with one and two altered profiles parameters (2, 5, and 7) [OR: 1.78, (95% CI:1.09–2.91), *P* < 0.021] and [OR: 1.67, (95% CI:1.04–2.71), *P* < 0.035], [OR: 1.69, (95% CI:1.21–2.36), *P* < 0.002]. In girls, the probability of having hyperglycemia was significantly associated with borderline-high LDL-C, with only one parameter from the altered lipid profile (profile 2) [OR: 1.46 (95% CI: 1.17–1.99), *P* < 0.017]. Consequently, it did not appear as a significant risk factor for variations in lipid profile variables.

**Table 3:** Risk factor for combined change in different profiles in increasing order.

Profile	TG	LDL	HDL	WC 90th percentile		Elevated BP		Elevated glucose	
				All participants					
				Odds ratio (95% CI)	*P*-value	Odds ratio (95% CI)	*P*-value	Odd ratio (95% CI)	*P*-value
1	D	D	D	1		1		1	
2	D	B	D	1.21 (0.78–1.88)	0.38	1.39 (1.08–1.80)	0.01	1.51 (1.16–1.96)	0.002
3	B	D	D	1.13 (0.48–2.61)	0.78	1.68 (1.06–2.66)	0.03	1.25 (0.86–1.83)	0.23
4	D	D	L	2.15 (1.40–3.28)	<0.001	1.62 (1.25–2.11)	<0.001	0.93 (0.78–1.11)	0.43
5	D	B	L	2.93 (1.96–4.37)	<0.001	2.04 (1.59–2.62)	0.007	1.13 (0.85–1.51)	0.39
6	B	B	D	3.78 (2.21–6.44)	<0.001	1.72 (1.16–2.55)	<0.001	1.74 (0.96–3.17)	0.07
7	B	D	L	3.53 (2.21–5.63)	<0.001	1.78 (1.30–2.44)	<0.001	1.53 (1.21–1.92)	<0.001
8	B	B	L	5.32 (3.42–8.27)	<0.001	2.45 (1.80–3.32)	<0.001	1.35 (0.88–2.06)	0.17
Boys									
1	D	D	D	1		1		1	
2	D	B	D	1.71 (0.59–5.0)	0.32	1.24 (0.78–1.95)	0.36	1.78 (1.09–2.91)	0.021
3	B	D	D	1.5 (0.17–13.2)	0.72	2.18 (0.94–5.06)	0.07	1.38 (0.67–2.84)	0.39
4	D	D	L	2.59 (0.97–6.98)	0.06	1.62 (1.05–2.47)	0.027	1.06 (0.80–1.39)	0.70
5	D	B	L	4.42 (1.68–11.58)	0.003	2.12 (1.38–3.25)	<0.001	1.67 (1.04–2.71)	0.035
6	B	B	D	5.02 (1.54–16.31)	0.007	2.15 (1.15–4.01)	0.02	1.81 (0.71–4.60)	0.21
7	B	D	L	3.61 (1.26–10.3)	0.02	1.79 (1.10–2.93)	0.019	1.69 (1.21–2.36)	0.002
8	B	B	L	9.95 (3.75–26.38)	<0.001	2.57 (1.58–4.17)	<0.001	1.49 (0.77–2.88)	0.23
Girls									
1	D	D	D	1		1		1	
2	D	B	D	1.13 (0.39–2.42)	0.62	1.46 (1.07–2.0)	0.02	1.46 (1.17–1.99)	0.017
3	B	D	D	0.97 (0.39–2.42)	0.95	1.38 (0.79–2.39)	0.25	1.25 (0.80–1.94)	0.32
4	D	D	L	2.21 (1.37–3.55)	0.001	1.77 (1.27–2.47)	<0.001	0.82 (0.66–1.03)	0.09
5	D	B	L	2.73 (1.76–5.74)	<0.001	2.10 (1.54–2.85)	<0.001	0.95 (0.66–1.36)	0.78
6	B	B	D	3.15 (1.73–5.74)	<0.001	1.28 (0.77–2.15)	0.34	1.66 (0.76–3.63)	0.20
7	B	D	L	3.57 (2.1–6.1)	<0.001	1.67 (1.09–2.54)	0.02	1.25 (0.89–1.74)	0.19
8	B	B	L	3.85 (2.31–6.41)	<0.001	2.17 (1.46–3.21)	<0.001	1.24 (0.71–2.16)	0.46

Data presented Odd ratio 95% CI; significant at *p* < 0.05; D, desirable; B, borderline/High; L, low.

## 4 Discussion

Dyslipidemia often initiates during adolescence and can persist into adulthood. Therefore, it is essential to identify dyslipidemia early in life, as timely intervention may mitigate the related morbidity and mortality associated with atherosclerosis and CVD in later years. Our present study demonstrated that approximately 44% of adolescents have at least one altered lipid profile variable and an increase in BMI and BMI z- score is associated with more significant alterations in lipid profile parameters, regardless of gender. In both boys and girls, the significant independent risk factors for combined dyslipidemia included WC and HTN. However, hyperglycemia was identified as a notable risk factor exclusively in boys.

Childhood obesity imposes a significant strain on the healthcare system. Research indicates that overweight and obesity often remain consistent from birth, continuing through childhood and adolescence into adulthood (25). Consequently, the prevalence of obesity in childhood is likely to adversely affect the health of future populations. Efforts to prevent childhood obesity will play a crucial role in mitigating the risk of related health issues, including among others dyslipidemia and type 2 diabetes (26).

A research study conducted in California, USA, gathered anthropometric data from individuals beginning at ages 5, 9–11, and/or 15–17 years, with follow-up assessments occurring around age 50 to evaluate anthropometric outcomes. Although, the study did not assess and compared lipid parameters, the findings revealed that at age 50, individuals who were classified as obese at age 5 exhibited BMI scores that were 6.51 units greater [95% CI = 3.67–9.35] compared to those who maintained a normal weight at the same age (27). Another longitudinal cohort study (age 11–18 years) with 24 year follow up indicates that high BMI during adolescence serves as a significant and independent risk factor for self-reported poor health, type 2 diabetes, hypertension, hyperlipidemia and early myocardial infarction in their younger adult age (30s and 40s) (28).

Current scientific evidence has proven that atherosclerosis is a process that begins in childhood tracks into adulthood culminating in atherosclerotic events (7). A recent prospective cohort study, which followed participants from the International Childhood Cardiovascular Cohort (i3C) Consortium over a span of 35 years, revealed that childhood risk factors and the variation in the combined-risk z score from childhood to adulthood were linked to cardiovascular events occurring in midlife. The analysis encompassed body mass index, systolic BP, total cholesterol levels, triglyceride levels, and smoking behaviors during youth (29). When dyslipidemia is identified and managed early in life, it can significantly decrease the likelihood of early cardiovascular complications and mortality (9).

The present study demonstrated at least one altered lipid profile parameter among majority of the Saudi adolescents (46.5%). Previous study from Saudi Arabia shows the overall prevalence of 25.5% dyslipidemia among adolescents with at least one abnormal lipid level (16). While comparing with other parts of the world, this prevalence was lower than Brazilian (64.7%), Ethiopian (63.9%), and Indian (77%), and higher than of Korean adolescents (19.7%) (30–33). The possible explanation for this variation may be due to differences in genetics, lifestyle factors and dietary habits (34). Moreover, racial/ethnic differences are related with variation in dyslipidemia (high triglyceride/Low high-density lipoprotein cholesterol) that could in turn affect racial/ethnic differences in cardiovascular disease (35). The present study shows the combination of two altered lipid levels including borderline/high TG and low HDL-C among adolescents with higher means of BMI and BMI-z score. This result is consistent with Brazilian study showing the most prevalent combination of dyslipidemia among adolescents as high TG with low HDL-C levels (30). Furthermore, high triglyceride levels present further risks for cardiovascular disease events, especially when they are coupled with low levels of HDL-C (36). Previous reports suggested that elevated childhood triglyceride or total cholesterol (TC) levels may be linked to risk factors contributing to CVDs in adulthood (37). Korean children and adolescents demonstrated a rise in mean LDL-C levels over time, along with a higher prevalence of elevated TG (greater than 110 mg/dL) and reduced HDL-C levels (less than 40 mg/dL) (38). Likewise, gender based high hypertriglyceridemia and low HDL-C were reported among Ethiopian children and adolescents (31).

Our present finding shows that irrespective of gender, there is an increase in alteration of lipid profile parameters with an increase in BMI. This finding is corroborated with study performed by Kaestner et al., demonstrating an increase in dyslipidemia with increasing BMI among Brazilian adolescents 30. Moreover, the occurrence of dyslipidemia rises alongside both an increase in BMI and advancing age showing at least one unfavorable lipid level with no significant differences found in the frequency of dyslipidemia in boys and girls (39). Our current study demonstrated WC and HTN as strong risk factors associated with increased altered lipid profile parameters for both boys and girls. Lee and colleagues demonstrated a positive association of elevated WC to increased levels of total cholesterol and LDL -C during middle to late adolescence in boys. Conversely, in girls, a high WC has been found to correlate with elevated total cholesterol, increased TG, and reduced HDL-C across early, middle, and late adolescence (40). Likewise, a significant relationship between BMI, WC, and the waist-to-height ratio (WHtR) with increased levels of LDL-C, TG, and TC was observed in individuals aged 6–18 years (41). Moreover, the combined presence of obesity, HTN, and dyslipidemia significantly elevates the risk of future cardiovascular incidents, such as myocardial infarction and stroke, among adolescents (42). Similarly, a recent study conducted in Sudan indicated that adolescents with higher age and BMI are at an increased risk of developing hypertension (43).

It has been widely noted that dyslipidemia is one of the metabolic disorders commonly associated with DM (elevated glucose levels) (6). There is a correlation between high glucose levels and factors such as age, gender, and an irregular lipid profile (44). However, the relationship between high glucose levels and their association with different lipid profiles in relation to gender has yielded conflicting results. Adolescent girls diagnosed with Type 1 Diabetes (T1D) have been observed to exhibit elevated mean levels of TG, and LDL-C, while their HbA1c levels do not significantly differ from those of boys (45). In addition, girls with T1D are shown to have higher odds of having CVD risk than boys, while another study found that boys were linked to a greater likelihood of progression in HDL-C levels (46, 47). The present study reinforces one of our previous findings that boys with high glucose levels are at a greater risk of having an altered lipid profile. Conversely, the relationship between elevated glucose levels and lipid profiles in girls was found to be less pronounced and not statistically significant (48).

Around 50% of adolescents with obesity exhibit at least one cardiovascular risk factor, while 10% present with three or more such factors, which encompass combined dyslipidemia, HTN, and insulin resistance (IR) (5, 6). Selective screening criteria may overlook the diagnosis of 30% to 60% of children with dyslipidemia. Consequently, it is advisable to implement a universal opportunistic screening approach for children between the ages of 9 and 11, as well as following the completion of pubertal development, specifically between 17 and 21 years of age (49). Similarly, the National Heart, Lung, and Blood Institute (NHLBI) guidelines indicate that screening should not be conducted for children aged 12–16 years, as they may yield inaccurately low results due to reduced lipid synthesis (50). It is therefore crucial to assess the lipid profiles of all children and adolescents who display signs or symptoms of dyslipidemia. Additionally, it is important to develop organized health management strategies that consider risk factors, with the goal of preventing dyslipidemia in this age group (51).

Early identification and intervention for pediatric dyslipidemia can significantly lower the risk of cardiovascular events and mortality. Additionally, as rates of pediatric obesity continue to rise, it is imperative to acknowledge and address dyslipidemia as a critical health concern in children and adolescents. The Saudi Vision 2030 is designed to reduce both the clinical and economic effects of CVD, with focus on prevention at various levels including individual educational and awareness efforts, community programs, and well-established primary care clinics that are easily accessible and provide high-quality screening and management services (52). The Saudi Health Council (SHC) has taken the concerning situation of dyslipidemia in Saudi patients very seriously and has established a task force to formulate national guidelines for the management of dyslipidemia (53). Our present study indicates that a high BMI by itself may serve as a marker for the necessity of dyslipidemia screening, while the other considerable associated risk factors with dyslipidemia could be high WC, HTN and glucose level. The findings of our study could be useful for policymakers to promptly implement public health initiatives. These initiatives should include the goal of managing and controlling lipid abnormalities and associated risk factors among adolescents in Saudi Arabia.

The authors acknowledge several limitations. First, the use of secondary data across multiple cohorts may introduce some degree of heterogeneity and that unmeasured confounders (such as diet, physical activity, smoking, and socioeconomic status) cannot be entirely ruled out. However, it is important to note that all samples were drawn from the same school-based population, using similar random selection procedures, during the same season in 2010, 2015, and 2019. This consistency in sampling framework and timing helps to minimize potential variability between cohorts. Unfortunately, lifestyle and socioeconomic variables were not consistently available in the datasets. Second, the cross-sectional nature of the study precludes any conclusions regarding causality. Nonetheless, one of this study's strengths lies in its extensive sample size, which accurately reflects the adolescent population in Saudi Arabia. Furthermore, it is the first study to deliver a detailed and sex-specific analysis of various combinations of dyslipidemia, alongside previously recognized risk factors in this demographic.

## 5 Conclusion

The presence of an abnormal BMI indicative of overweight or obesity among Saudi adolescents is directly associated with changes in the lipid profile. It has also been demonstrated that the most prevalent altered combination between two modified lipid variables is that of borderline/high TG and low HDL-C, indicating a potential risk for Saudi adolescents. In addition, the probability of having greater altered lipid profile was strongly associated with high WC, and BP. However, elevated glucose level was strongly associated with altered lipid profile in boys as compared to girls. This study suggests BMI as a valuable indicator for diagnosing dyslipidemia in adolescents and support the strong association between lipid profiles, and other CVD risk factors such as high WC, HTN, and gender specific hyperglycemia level. In Saudi Arabia, the screening for CVD risk factors, including the lipid profile, ought to be conducted earlier than recommended for developed nations (53). Therefore, the present study recommends facilitating early interventions in Saudi adolescents with an aim for lipid management ought to consider not only the absolute lipid levels but also the overall cardiovascular risk profile.

## Data Availability

The original contributions presented in the study are included in the article/Supplementary Material, further inquiries can be directed to the corresponding author.
